# Development and application of a serious adverse events risk model for concurrent chemoradiotherapy in patients with nasopharyngeal carcinoma

**DOI:** 10.1097/MD.0000000000039377

**Published:** 2024-08-23

**Authors:** Jiahui Li, Qianwen Liu, Huiying Qin

**Affiliations:** a School of Nursing, Sun Yat-sen University, Guangzhou, Guangdong Province, China; b Department of Phase I Clinical Trials, Sun Yat-sen University Cancer Center, Guangzhou, Guangdong Province, China; c Department of Nursing, Sun Yat-sen University Cancer Center, Guangzhou, Guangdong Province, China.

**Keywords:** adverse event, concurrent chemoradiotherapy, nasopharyngeal cancer, risk model

## Abstract

The objective of this study was to construct a concise prediction model for serious adverse events (SAEs) in order to assess the likelihood of SAE occurrence among hospitalized patients undergoing concurrent chemoradiotherapy. An electronic database of a Cancer Centre was utilized to conduct a cross-sectional review survey. Our research involved the recruitment of 239 patients who were undergoing concurrent chemoradiotherapy in the Department of Nasopharynx and Radiotherapy. The clinical prediction rule was derived using logistic regression analysis, with SAE serving as the primary outcome. Internal verification was conducted. The occurrence rate of SAE in the derivation cohort was 59.4%. The ultimate model used had 3 variables, namely cystatin C, C-reactive protein, and serum amyloid A. The model exhibited an area under the curve of 0.626 (95% CI: 0.555–0.696; *P* < .001). The model accurately predicts the occurrence of SAE, and the variable data can be easily obtained, and the assessment technique is straightforward.

## 1. Introduction

The worldwide prevalence of nasopharyngeal carcinoma (NPC) is reported to be 133,354, resulting in 80,008 deaths.^[1]^ Approximately 80% of nasopharyngeal malignancies globally are concentrated in the southern region of China, resulting in an incidence rate of 52,000 cases and 267,000 deaths.^[2]^ The primary treatment for nasopharyngeal cancer is multidisciplinary therapy utilizing radiation.^[3]^ Nasopharyngeal cancer is frequently disregarded due to the absence of initial symptoms, resulting in the diagnosis of most patients with NPC occurring at an advanced stage within the local area.^[4]^ The current consensus is that the combination of induction chemotherapy and concurrent chemoradiotherapy (CCRT) provides a survival advantage for patients with locally progressed nasopharyngeal cancer.^[5]^ CCRT is frequently suggested as a therapeutic modality in guidelines pertaining to nasopharyngeal cancer.^[6,7]^

The administration of chemotherapy has been shown to enhance the sensitivity of radiotherapy and disrupt the process of repairing sublethal damage to tumor cells. This interference serves to improve local control and decrease the occurrence of distant metastases.^[8]^ Nevertheless, the lack of specificity in sensitization of chemotherapeutic medicines might lead to radiotoxicity in patients and the occurrence of novel adverse effects.^[9]^ Multiple clinical trials have identified various negative occurrences that take place during CCRT, such as leukopenia, myelosuppression, mouth ulcers, gastrointestinal problems, and others.^[10,11]^ The efficacy of chemotherapy and radiotherapy may be considerably diminished in cases when patients experience heightened levels of adverse responses. In certain conditions, treatment may be halted.^[12,13]^

Adverse events are categorized into several degrees according on their severity, as determined by the Common Terminology Criteria for Adverse Events v5.0 (CTCAE5.0) (NCI, 2017) scores. Specifically, adverse events with scores below 3 are classed as significant adverse events (SAEs). SAE result in physical distress, diminished adherence to therapy and overall well-being, and escalated healthcare expenses.^[14,15]^ Hence, assessing the likelihood of SAE is a crucial component of CCRT in the management of NPC. Prior research on the characteristics that affect SAE in CCRT for patients with NPC mostly examined biochemical markers, patients’ overall health status, and treatment protocols.^[16–18]^ The practicality of the influencing elements for clinical practice in assessing the overall level of danger to patients is insufficient. There exists a pressing need to develop a user-friendly and straightforward instrument for evaluating SAE in the context of concurrent radiation for individuals diagnosed with nasopharyngeal cancer.

Risk prediction models are utilized to estimate the likelihood of an undesirable event occurring in a person by including a variety of risk prediction outcomes.^[19]^ An effective predictive model has the ability to differentiate between a cohort of individuals with a high and low likelihood of experiencing a specific result. Therefore, a scientific and efficient model for predicting the likelihood of SAEs can guarantee the safety of patients and direct a more suitable emphasis on preventing SAEs. Hence, our objective was to construct an SAE risk model for hospitalized patients with NPC undergoing CCRT treatment. This model would enable us to promptly identify the risk variables associated with SAE and implement proactive interventions to reduce the occurrence of SAE and enhance the patients’ quality of life.

## 2. Material and methods

### 2.1. Patient identification and eligibility

An electronic database of the Cancer Centre of Sun Yat-sen University was utilized to conduct a cross-sectional review survey. The participants included in the research were patients diagnosed with NPC who were admitted to the Department of Nasopharynx and Radiotherapy during the period from September 1, 2020, to August 30, 2021. The study subjects were selected based on specific inclusion criteria. These criteria included the confirmation of NPC through pathologic biopsy prior to treatment for the first time, absence of any history of cancer treatment, absence of a second malignancy, complete medical records in the database, all patients receiving concurrent chemotherapy consisting of 2DRT/3DCRT and platinum-based chemotherapy, and nonparticipation in any other clinical trials. According to the 8th edition of the American Joint Commission on Cancer TNM staging standards, all patients were categorized. The Sun Yat-sen University Cancer Center (GYX2019-020) granted ethical approval from its Institutional Review Boards. The Institutional Review Boards did not mandate patients’ agreement to review their medical records, as the study solely relied on the examination of their files, and the confidentiality of patient data was upheld. The study adheres to the guidelines outlined in the 1995 Declaration of Helsinki (as amended in Edinburgh in 2000).

### 2.2. Baseline predictor variables and outcome measures

The clinical data of 239 patients were analyzed, including biochemical indicators, patients’ general condition, and treatment regimens within 24 hours before CCRT. The variables recorded included from hospitalized patients were sex, age, body mass index, induction chemotherapy regimen, CCRT regimen, American Joint Commission on Cancer TNM staging, age-adjusted Charlson Comorbidity Index, Nutritional Risk Screening 2002 (NRS2002), aspartate aminotransferase, glutamic-pyruvic transaminase, creatinine, cystatin C (Cys C), C-reactive protein (CRP), serum amyloid A (SAA), white blood cell count, neutrophils, lymphocytes, hemoglobin, and platelet count/blood platelet count. Hematological indicators are classified according to the reference interval of WS/T 404.4-2018 Reference Intervals for Clinical Standing Test Items promulgated by the National Health Care Commission of the People’s Republic of China. Body mass index was categorized by World Health Organization. The scales of NRS2002 and age-adjusted Charlson Comorbidity Index are classified according to their cutoff values. Patients with SAE (CECTA 5.0 ≥ 3 grades) during hospitalization were included in the SAE group; otherwise, they were included in the non-SAE group.

### 2.3. Statistical analyses

The statistical analysis was conducted using SPSS 25.0 Statistics (IBM, Chicago, IL) and R software version 4.1.2. The sole purpose of utilizing the R software was to assess the Akaike information criterion (AIC). The mean and standard deviation were used to characterize continuous data. A *t* test was employed to compare groups. The statistical tests employed in this study were the chi-square and Mann–Whitney *U* tests. The data was characterized using frequency and components ratio or percentage. The researchers created a logistic regression model in which SAE was included as an independent variable. Multivariable logistic regression was used to identify risk variables for SAE based on components with a single-factor logistic regression *P*-value < .10. The logistic regression equation was used to calculate the projected risk of SAE for each patient in the validation pool by substituting patient data. Probability prediction was conducted using the receiver operating characteristic (ROC) curve, and internal validation was carried out using bootstrap methods. The Hosmer–Lemeshow test was utilized to assess the calibration degree of the logistic regression model by comparing the predicted probability of SAE with the actual incidence of SAE. The dataset comprised patients with nasopharyngeal carcinoma undergoing concurrent chemoradiotherapy. Data were randomly allocated into a development set (70%) and a validation set (30%). Using the development set, a logistic regression model was constructed to identify risk factors for SAE, selecting predictors based on univariate analyses with a significance threshold of *P* < .10. The logistic regression equation calculated the projected risk of SAE for each patient.

## 3. Results

### 3.1. General conditions in the SAE group and the non-SAE group

A total of 239 patients were enrolled in the study, with 194 (81.2%) males and 45 (18.8%) females. One hundred forty-two patients experienced SAE (59.4%) during concurrent chemoradiotherapy, and the most common SAE was oral mucositis (43.1%). The results of ALB, Cys C, CRP, and SAA were significantly different between the 2 groups (*P* < .05) (Table 1).

**Table 1:** Comparison of the characteristics of the SAE and non-SAE groups.

Patient characteristics	SAE group	Non-SAE group	t or Z value	*P*-value	Odds ratio, 95% CI confidence interval
Sex					
Male	83 (85.6%)	111 (78.2%)	1.481	.14	1.66 (0.83, 3.31)
Female	14 (14.4%)	31 (21.8%)			
Age (years)					
<60	88 (90.7%)	131 (92.3%)	−0.418	.676	0.82 (0.33, 2.06)
≥60	9 (9.3%)	11 (7.7%)			
aCCI					
<3	53 (52.1%)	74 (52.1%)	0.383	.702	1.11 (0.66, 1.86)
≥3	44 (45.4%)	68 (47.9%)			
TNM stage†					
II/III	55 (56.7%)	79 (55.6%)	0.163	.871	1.04 (0.62, 1.76)
IV	42 (43.3%)	63 (44.4%)			
IC regimen					
No	17 (17.5%)	25 (17.6%)	−0.016	.987	1.00 (0.51, 1.96)
Yes	80 (82.5%)	117 (82.4%)			
CCRT regimen					
CCRT alone	75 (77.3%)	99 (69.7%)	1.318	.189	1.48 (0.82, 2.68)
CCRT + immunotherapy/targeted therapy	22 (22.7%)	43 (30.3%)			
BMI (kg/m^2^)					
18.5–24.0	55 (56.7%)	76 (53.5%)	0.483	.629	1.14 (0.68, 1.91)
<18.5/>24.0	42 (43.3%)	66 (46.5%)			
NRS2002					
<3	53 (54.6%)	74 (52.1%)	0.383	.702	1.11 (0.66, 1.86)
≥3	44 (45.4%)	68 (47.9%)			
ALT (U/L)					
≤40	91 (93.8%)	137 (96.5%)	−0.963	.336	0.55 (0.16, 1.87)
>40	6 (6.2%)	5 (3.5%)			
AST (U/L)					
≤35	93 (95.9%)	140 (98.6%)	−1.202	.231	0.33 (0.06, 1.85)
>35	4 (4.1%)	2 (1.4%)			
ALB (g/L)					
≥40	97 (100.0%)	132 (93.0%)	3.268	.001*	0.58 (0.52, 0.64)
<40	0 (0.0%)	10 (7.0%)			
CRE (μmol/L)					
≤73	57 (58.8%)	73 (51.4%)	1.122	.263	1.35 (0.80, 2.27)
>73	40 (41.2%)	69 (48.6%)			
Cys C (mg/L)					
≤1.03	74 (76.3%)	86 (60.6%)	2.629	.009*	2.10 (1.18, 3.73)
>1.03	23 (23.7%)	56 (39.4%)			
CRP (mg/L)					
≤3	82 (84.5%)	94 (66.2%)	3.377	.001*	2.79 (1.46, 5.35)
>3	15 (15.5%)	48 (33.8%)			
SAA (mg/L)					
≤10	79 (81.4%)	98 (69.0%)	2.236	.026*	1.97 (1.06, 3.68)
>10	18 (18.6%)	44 (31.0%)			
WBC (109/L)					
3.5–9.5	63 (64.9%)	103 (72.5%)	−1.233	.219	0.70 (0.40, 1.22)
<3.5/>9.5	34 (35.1%)	39 (27.5%)			
NE (109/L)					
3.5–9.5	58 (59.8%)	96 (67.6%)	−1.226	.222	0.71 (0.42, 1.22)
<3.5/>9.5	39 (40.2%)	46 (32.4%)			
LY (109/L)					
1.1–3.2	76 (78.4%)	117 (82.4%)	−0.776	.438	0.77 (0.40, 1.48)
<1.1/>3.2	21 (21.6%)	25 (17.6%)			
HGB (g/L)					
<130	36 (37.1%)	44 (31.0%)	0.984	.326	1.31 (0.76, 2.27)
≥130	61 (62.9%)	98 (69.0%)			
PLT (109/L)					
125–350	79 (81.4%)	120 (84.5%)	−0.621	.535	0.81 (0.41, 1.60)
<125/>350	18 (18.6%)	22 (15.5%)			

aCCI = age-adjusted Charlson Comorbidity Index, ALT = glutamic-pyruvic transaminase, AST = aspartate aminotransferase, BMI = body mass index, CCRT = concurrent chemoradiotherapy regimen, CI = confidence interval, Cr = creatinine, CRP = C-reactive protein, Cys C = cystatin C, HGB = haemoglobin, IC = induction chemotherapy, LY = lymphocytes, NE = neutrophils, NRS2002 = Nutritional Risk Screening 2002, PLT = platelet count/blood platelet count, SAA = serum amyloid A, WBC = white blood cell count. BMI = weight (kg)/height (m^2^).

† TNM stage was defined by criteria of the 8th edition of the American Joint Commission on Cancer (AJCC).

* *P* < .05.

### 3.2. Results of logistic regression analysis

Univariate logistic regression factors with a *P*-value < .10 were qualified for multivariable logistic regression. The final factors entered into the model were Cys C, CRP, and SAA. The model was statistically significant (likelihood ratio chi-square = 15.034, degree of freedom = 3, and *P* = .002), and the AIC was 315.77. The AIC without the SAA indicator was 314.10. Cys C > 1.03 mg/L, CRP > 3 mg/L, and SAA > 10 mg/L were all the risk factors for SAE (Table 2).

**Table 2:** Multivariable logistic regression analysis of multiple factors.

	Regression coefficient (*β*)	SE of regression coefficient	Wald *χ*^2^	*P*-value	Odds ratio, 95% CI
Constant	−0.053	0.176	0.089	.765	-
Cys C > 1.03 mg/L	0.594	0.303	3.838	.050	1.811 (1.000, 3.280)
CRP > 3 mg/L	0.832	0.373	4.972	.026	2.298 (1.106, 4.774)
SAA > 10 mg/L	0.210	0.364	0.335	.563	1.234 (0.605, 2.518)

CI = confidence interval, CRP = C-reactive protein, Cys C = cystatin C, SAA = serum amyloid A, SE = standard error.

### 3.3. Internal verification of logistic regression model

**Figure 1.**
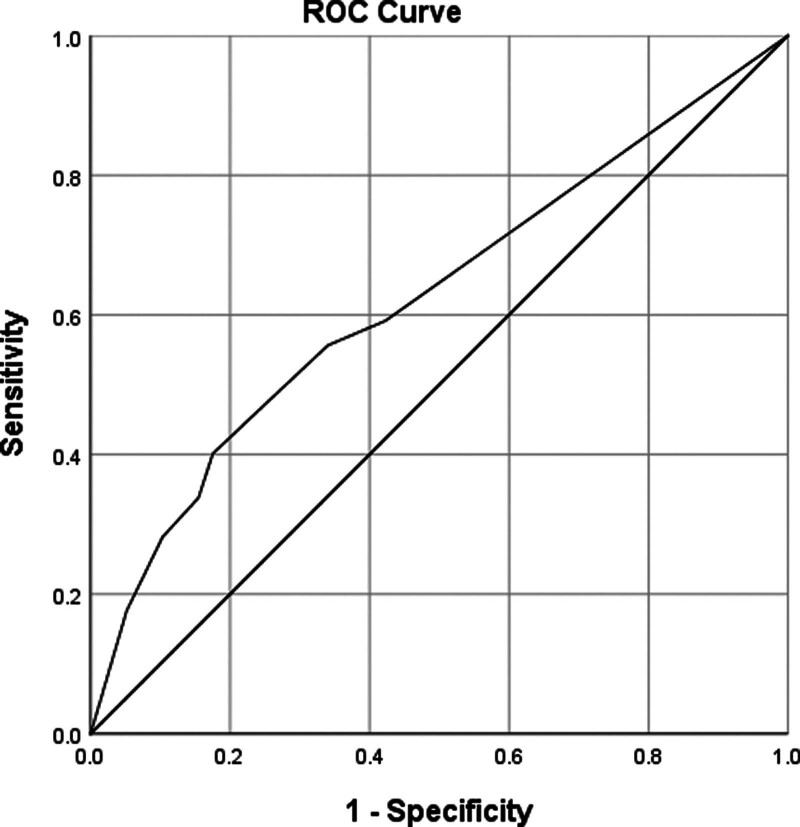
ROC curve from internal validation of the logistic regression model for predicting serious adverse events (SAEs), with an AUC of 0.626.

Substituting the patient data into the logistic regression formula above, the probability of SAE was 48.7% to 83.0% and the risk of SAE was classified as “low” and “high” according to the probability of occurrence (Table 3). Predicted probabilities were analyzed using ROC curves (Fig. 1), with an area under the curve (AUC) = 0.626 (95% CI: 0.555–0.696; *P* < .001); the predicted probability of the ROC curve without the SAA indicator was (95% CI: 0.557–0.698; *P* < .001). The differences were both statistically significant. In the 1000 bootstrap datasets, the AUC was 0.642; the model was not over fitted. Based on the predicted probability and the actual occurrence of SAE, the *P*-value of the Hosmer–Lemeshow goodness-of-fit statistic with 4 degrees of freedom was 0.717, and the chi-square was 2.104. The prediction of SAE was in good accordance with the actual occurrence.

**Table 3:** Probability and risk level of SAE based on Cys C, CRP, and SAA.

Cys C (mg/L)	CRP (mg/L)	SAA (mg/L)	Probability of SAE (%)	Risk level
≤1.03	≤3	≤10	48.7%	Low
≤1.03	≤3	>10	53.9%	High
>1.03	≤3	≤10	63.2%	High
>1.03	≤3	>10	67.9%	High
≤1.03	>3	≤10	68.6%	High
≤1.03	>3	>10	72.9%	High
>1.03	>3	≤10	79.8%	High
>1.03	>3	>10	83.0%	High

CRP = C-reactive protein, Cys C = cystatin C, SAA = serum amyloid A.

### 3.4. External validation of the logistic regression model

**Figure 2.**
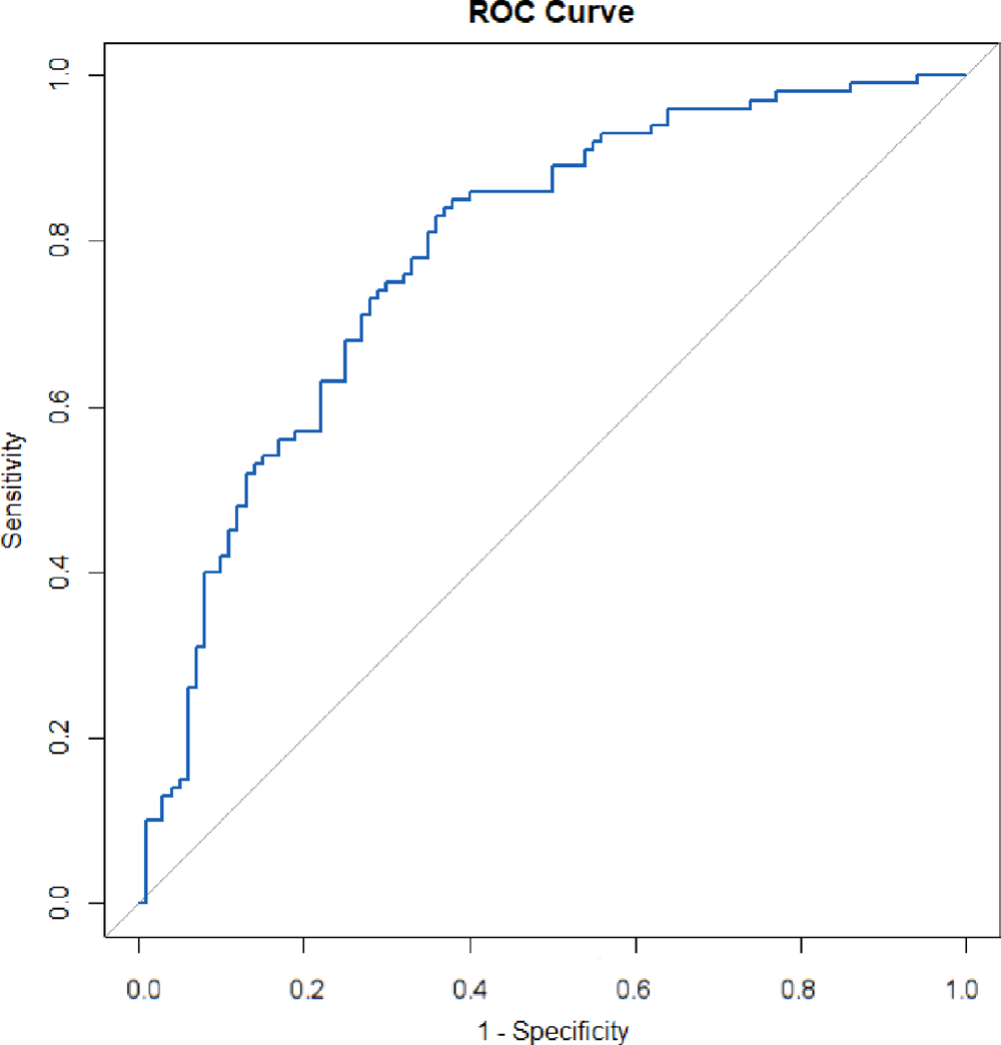
ROC curve from external validation of the logistic regression model, showing improved predictive accuracy with an AUC of 0.784.

The dataset comprised 239 patients with nasopharyngeal carcinoma undergoing concurrent chemoradiotherapy. Data were randomly allocated to a development set, consisting of approximately 70% (n = 167) of the patients, and a validation set, comprising the remaining 30% (n = 72). The logistic regression model was applied to this separate validation set. Predicted probabilities of SAE were analyzed using ROC curves. Despite some variability, the model exhibited an AUC of 0.784 in the validation set, demonstrating good predictive ability and significantly improved discrimination compared to earlier estimates (Fig. 2). The Hosmer–Lemeshow test yielded a chi-square of 3.87 with a *P*-value of .423, indicating a satisfactory fit between the predicted and observed outcomes. This suggests that the model adequately captures the underlying risk factors and can be used reliably in clinical settings to predict SAE occurrences among patients undergoing concurrent chemoradiotherapy for nasopharyngeal carcinoma.

### 3.5. Rules for predicting SAE risk based on the model

Depending on the model, rules were developed for predicting SAE risk (Table 4), with risk rated as “low risk” if 3 evaluations resulted in “no.” If one of the predictors is evaluated as “yes,” the risk is rated as “high risk.”

**Table 4:** SAE risk assessment form for CCRT of NPC.

	Yes	No
Cys C > 1.03 mg/L	□	□
CRP > 3 mg/L	□	□
SAA > 10 mg/L	□	□
SAE risk	□High	□Low

In this table, checkboxes (□) are used to indicate whether each parameter is above the specified threshold, influencing the assessment of SAE risk.

CRP = C-reactive protein, Cys C = cystatin C, SAA = serum amyloid A, SAE = serious adverse events.

## 4. Discussion

We created a clinical prognostic algorithm to assess the likelihood of SAE in patients with NPC who underwent CCRT treatment. This model is the initial predictive model for SAE during CCRT for NPC, as far as we know. Our model’s internal validation demonstrated strong performance in identifying SAE events, particularly in accurately predicting clinical significance. The predictive tool demonstrated a moderate level of accuracy. The area under the ROC curve is 0.626, indicating a 62.6% likelihood of accurately identifying patients at risk of SAE. The probability of SAE exhibited a progressive increase with the inclusion of 3 independent variables, namely Cys C > 1.03 mg/L, CRP > 3 mg/L, and SAA > 10 mg/L. The laboratory results, which were incorporated into the majority of the early tests, constituted the final components in the model.^[20]^

The prevalence of SAE in hospitalized patients with NPC undergoing CCRT was determined to be 59.4%. Oral mucositis was the most prevalent serious adverse event by 43.1%. The findings of Lv et al^[17]^ were consistent with this, indicating that the occurrence of SAE was 63%, with mucositis being the most prevalent grade 3 to 4 adverse event at 41%. The occurrence of SAE was primarily observed during the second cycle of concomitant radiation, and it was found to be associated with a cumulative dosage of chemoradiotherapy toxicity.^[20]^ Acute toxic events are an inevitable consequence of high dose chemoradiotherapy. Nevertheless, it is important to note that grade 3 or 4 events might persist and result in significant late consequences.^[21]^ Hence, conducting regular risk assessments on patients for SAE enables healthcare practitioners to promptly identify individuals who are at risk and furnish crucial information for tailored therapy.

The inflammatory variables that served as autonomous predictors in our investigation were CRP and SAA. The phenomenon of inflammation is intricately linked to tissue damage and infection, as it has the ability to modify the microenvironment of tumors and play a role in their invasion, migration, and metastasis.^[22,23]^ The prognosis of human malignancies has been found to be related with many inflammation-based scores, including the neutrophil-to-lymphocyte ratio.^[24,25]^ Furthermore, inflammatory markers are cost-effective and very feasible.^[26,27]^ Through the evaluation of the patient’s indications mentioned earlier, physicians can select a customized treatment strategy for NPC patients and provide them with information regarding their management.

CRP > 3 mg was a high-risk factor in the predictive rule for SAE of CCRT in NPC patients. The finding was similar to conclusions reached by Tominaga et al^[28]^ (CRP as a significant determinant of SAE). Nevertheless, in a study by Han et al,^[8]^ SAE in patients with NPC was not associated with CRP, but only with gender, T stage, N stage, and treatment plan. The utility of CRP on the prognosis of nasopharyngeal carcinoma requires further study. However, as a component of the innate immune system, CRP has been regarded as a valuable inflammatory marker in cardiovascular disease, chronic hepatitis, and so on.^[29]^ A systematic evaluation of 271 articles showed that high CRP was associated with higher mortality and poor treatment response in 90% of patients with primary solid tumors.^[30]^ In clinical practice, it is still prudent for healthcare professionals to consider changes in CRP during treatment for patients’ safety. Higher pretreatment levels of serum CRP may also identify candidates for more aggressive monitoring and individualized treatment.

Furthermore, SAA levels exceeding 10 mg/L were identified as an additional autonomous risk factor for SAE of CCRT in NPC patients. Several studies have demonstrated that SAA has the potential to serve as a predictive biomarker for various types of cancer and plays a crucial role in determining SAE in NPC.^[31,32]^ SAA has the ability to induce the expression of MMP-9 in macrophages, which subsequently facilitates the spread of cancer cells.^[33]^ In the interim, it has been suggested that SAA could potentially play a role in the advancement of cancer by impeding platelet adhesion and enhancing fibrinogen activation.^[34]^ In the present investigation, it was observed that elevated blood SAA levels were associated with an increased risk of SAE alone in the univariate analysis. However, this association did not reach statistical significance in the multifactor analysis (*P* = .563). The models were assessed individually, both with and without SAA, and the resulting model fits did not exhibit any significant differences (AIC = 314.10, 315.77; ROC = 0.627, 0.626). The inclusion of SAA as a risk factor for SAE in our investigation was motivated by the clinical significance of the SAA indication, which may be attributed to the relatively small sample size of the study. Additional research, particularly extensive prospective studies, is required to establish that SAA is a valuable prognostic biomarker that can offer supplementary insights for personalized cancer treatment.

Several studies have indicated that the overall condition and treatment protocols have an impact on the outcome of SAEs.^[35,36]^ Our findings contradict the association between patients’ treatment prognosis and indicators such as age and comorbidities.^[37,38]^ The potential cause for the observed outcomes could be attributed to the oversimplified categorization employed in the research. In light of the constraints imposed by limited research capability, our study solely provided an initial categorization of patients’ treatment alternatives, and regrettably, it did not yield any evidence supporting the efficacy of various treatment options.

Previous investigations have used a comprehensive set of hematological parameters, encompassing nutritional, liver function, renal function, and blood count indicators.^[5,32,38]^ Nevertheless, our investigation solely determined that Cys C levels exceeding 1.03 mg/L were indicative of a high-risk factor in predicting the likelihood of SAE. The measurement of serum cystatin C levels is of significant importance in the identification and prognostication of various human ailments, including renal illness and cardiovascular disease.^[5]^ Cys C levels are unaffected by age and gender, making it a valuable tool for monitoring renal function and assessing the toxicity of chemotherapy drugs in cancer patients.^[5,10]^ The careful monitoring of Cys C levels can be instrumental in implementing preventive strategies to mitigate the occurrence of SAE in patients with NPC at an early stage.^[23]^ In addition to the Cys C indicator, the research did not find statistical significance for other hematological indicators. This lack of significance could perhaps be attributed to variations in measurement time points and the presence of selection bias.

Following the internal validation of our model, which achieved an AUC of only 0.626 (95% CI: 0.555–0.696), indicating limited predictive power for SAEs, we conducted external validation. The dataset was randomly divided into a development set (70%, 167 patients) and a validation set (30%, 72 patients), allowing for robust model development and independent performance validation. In this validation, the model demonstrated strong predictive capability with an AUC of 0.784, accurately distinguishing between patients who did and did not experience SAEs. The low chi-square value and high *P*-value indicate a nonsignificant difference between predicted and observed outcomes, confirming the model’s good statistical fit. This indicates that the model appropriately captures the risk factors influencing the occurrence of SAEs. Given its proven predictive accuracy and satisfactory statistical fit, the model can reliably be used in clinical settings to predict the risk of SAEs in patients with nasopharyngeal carcinoma undergoing concurrent chemoradiotherapy. This predictive tool is crucial for clinical decision-making as it assists physicians in assessing treatment-related risks, thereby enabling more informed therapeutic decisions.

Our study is limited by the relatively small sample size and the use of single-center studies. The factors that influence SAE in hospitalized patients are intricate. Due to our restricted research capability, we were unable to examine immunological and genetic characteristics in patients before admission, which hindered our ability to extensively explore relevant variables and draw prediction criteria for SAE. In subsequent investigations, the inclusion of immunological and genetic variables may be considered to enhance the robustness of the risk model through further study. Furthermore, our study employed a very straightforward classification of variables. Future research endeavors could enhance the outcomes of variable stratification and conduct more comprehensive analysis of the variable results. The evaluation and analysis of SAE patterns and situations were not conducted in this study, as it is imperative to address every SAE with due seriousness and promptly intervene. Due to time constraints and the unavailability of a new external dataset, we implemented a pseudo-external validation approach. Although practical, this method may limit the model’s generalizability compared to traditional external validation.

## 5. Conclusion

We introduce a predictive model for assessing the probability of SAEs in patients with NPC who are undergoing concurrent chemoradiotherapy. The accessibility of variable data and the simplicity of the evaluation procedure make them valuable resources for preventing SAE and identifying patients who are at a heightened risk for SAE. The study findings suggest that it is crucial for practical clinical practice to thoroughly test patients’ Cys C, CRP, and SAA levels to rapidly diagnose patients’ symptoms and effectively manage SAE. Subsequent investigations will focus on verifying the risk model using external data in a more extensive group of patients with NPC, to aid in the development of specific therapies.

## Author contributions

**Conceptualization:** Jiahui Li.

**Formal analysis:** Qianwen Liu.

**Investigation:** Jiahui Li.

**Methodology:** Jiahui Li.

**Resources:** Qianwen Liu.

**Software:** Qianwen Liu.

**Supervision:** Huiying Qin.

**Visualization:** Huiying Qin.

**Writing – original draft:** Jiahui Li.

**Writing – review & editing:** Huiying Qin.
